# Thyroid diseases increased the risk of type 2 diabetes mellitus: A nation-wide cohort study

**DOI:** 10.1097/MD.0000000000015631

**Published:** 2019-05-17

**Authors:** Rong-Hsing Chen, Huey-Yi Chen, Kee-Ming Man, Szu-Ju Chen, Weishan Chen, Po-Len Liu, Yung-Hsiang Chen, Wen-Chi Chen

**Affiliations:** a Departments of Endocrine and Metabolism, Anesthesiology, Obstetrics and Gynecology, Medical Research, Medical Education, and Urology, China Medical University Hospital; b Graduate Institute of Integrated Medicine, College of Chinese Medicine, College of Medicine, China Medical University; c Department of Anesthesiology, China Medical University Hsinchu Hospital, Hsinchu; d Department of Surgery, Taichung Veterans General Hospital, Taichung; e Management Office for Health Data, China Medical University Hospital, Taichung; f Department of Respiratory Therapy, College of Medicine, Kaohsiung Medical University, Kaohsiung; g Department of Psychology, College of Medical and Health Science, Asia University, Taichung, Taiwan.

**Keywords:** hyperthyroidism, hypothyroidism, nation-wide cohort study, thyroid disease, type 2 diabetes mellitus

## Abstract

Thyroid function may alter carbohydrate metabolism via influence of insulin, which may in terms of derangement of thyroid function and insulin function result in the development of type 2 diabetes mellitus (T2D). We investigated the association of thyroid disorders with T2D by a cohort study of the Taiwan nationwide health insurance database.

A sub-dataset of the National Health Insurance Research Database (NHIRD) was used in this study. The thyroid disease (both hyper- and hypo-thyroidism) group was chosen from patients older than 18 years and newly diagnosed between 2000 and 2012. The control group consisted of randomly selected patients who never been diagnosed with thyroid disease and 4-fold size frequency matched with the thyroid disease group. The event of this cohort was T2D (ICD-9-CM 250.x1, 250.x2). Primary analysis was performed by comparing the thyroid disease group to the control group and the second analysis was performed by comparing the hyperthyroidism subgroup, hypothyroidism subgroup, and control group.

The occurrence of T2D in the thyroid disease group was higher than the control group with hazard ratio (HR) of 1.23 [95% confidence interval (CI) = 1.16–1.31]. Both hyperthyroidism and hypothyroidism were significantly higher than control. Significantly higher HR was also seen in female patients, age category of 18 to 39-year-old (y/o) and 40 to 64 y/o subgroups. Higher occurrence of T2D was also seen in thyroid disease patients without comorbidity than in the control group with HR of 1.47 (95% CI = 1.34–1.60). The highest HR was found in the half-year follow-up.

There was a relatively high risk of T2D development in patients with thyroid dysfunctions, especially in the period of 0.5 to 1 year after presentation of thyroid dysfunctions. The results suggest performing blood sugar tests in patients with thyroid diseases for early detection and treatment of T2D.

## 1 Introduction

Diabetes mellitus (DM) is a very common disease with a global prevalence rate of 8.5% in adult subjects, most of which are type 2 DM (T2D).^[1–3]^ T2D is due to insulin resistance associated with insulin deficiency, which may result in carbohydrate derangement and hyperglycemia.^[4–6]^ Long-term hyperglycemia may cause various acute and chronic complications and is the leading cause of blindness, cardiovascular disease, renal failure, and even death.^[7]^ It is not only a big challenge in the clinical setting but also a heavy burden for public health.

Thyroid hormones are essential for carbohydrate metabolism.^[8–10]^ Hyperthyroidism (thyroid hormone excess) may affect the secretion, action, and clearance of insulin and many aspects of carbohydrate metabolism and thus lead to hyperglycemia.^[11–15]^ On the contrary, hypothyroidism (thyroid hormone deficiency) may also interfere with the action and metabolism of insulin and induce insulin resistance.^[16–18]^

Both hyperthyroidism and hypothyroidism are also common clinical disorders with similar prevalence rates of around 0.5% to 2%.^[19]^ Thyroid hormone can modulate carbohydrate metabolism; thus, in theory, thyroid dysfunctions may influence the development of DM. However, there is a lack of studies of the influence of thyroid dysfunctions on T2D occurrence, and existing studies are inconsistent.^[20–24]^ It is therefore necessary to further investigate the development of T2D after the presentation of both hyperthyroidism and hypothyroidism.

## 2 Methods

### 2.1 Data sources

The National Health Insurance (NHI) program of Taiwan was established in 1995; it covers more than 99% of Taiwan's population.^[25]^ The Longitudinal Health Insurance Database 2000 (LHID2000) was a sub-dataset of the National Health Insurance Research Database (NHIRD) that randomly selected 1 million patients from the NHIRD. We used this database in this study. We were able to obtain patient demographics, inpatient and outpatient records, medications, and treatments from the database. To protect the patient privacy, the identification number has been reencoded. This study was approved by the Institutional Review Board of China Medical University and Hospital Research Ethics Committee (permit number: CMUH-104-REC2–115).

### 2.2 Sampled participant

The thyroid disease group was selected from patients older than 18 years and newly diagnosed with thyroid diseases (ICD-9-CM 242–244) between 2000 and 2012. We then divided the thyroid disease group into 2 subgroups, the hyperthyroidism subgroup and hypothyroidism subgroup. Patients with hyperthyroidism (ICD-9-CM 242) belonged to the hyperthyroidism subgroup and patients with hypothyroidism (ICD-9-CM 243–244) were in hypothyroidism subgroup. Patients who had been diagnosed with hyperthyroidism and hypothyroidism were not included in our study. The index date was the date the patient was diagnosed with thyroid disease. We then excluded the patients who had history of DM (ICD-9-CM 250), neonatal diabetes (ICD-9-CM 775.0, 775.1), genetic defects (ICD-9-CM 259.8), pancreatitis (ICD-9-CM 577), pancreatectomy (ICD-9-CM 251.3), cystic fibrosis (ICD-9-CM 277.00), hemochromatosis (ICD-9-CM 275.0), pancreatic neoplasia (ICD-9-CM 577.8), acromegaly (ICD-9-CM 253.0), Cushing syndrome (ICD-9-CM 255.0), Glucagonoma (ICD-9-CM 157.4, 211.7), pheochromocytoma (ICD-9-CM 194.0, 227.0), drug induced diabetes (ICD-9-CM 790.2), rubella (ICD-9-CM 771.0), cytomegalovirus (ICD-9-CM 078.5, 771.1), Stiff-man syndrome (ICD-9-CM 333.91), other genetics (ICD-9-CM 364.61), Klinefleter syndrome (ICD-9-CM 758.7), Turner syndrome (ICD-9-CM 758.6), Friedreich ataxia (ICD-9-CM 334.0), Huntington chorea (ICD-9-CM 333.4), Laurence–Moon–Biedl syndrome (ICD-9-CM 759.89), myotonic dystrophy (ICD-9-CM 359.2), porphyria (ICD-9-CM 277.1), Prader-Willi syndrome (ICD-9-CM 759.81), and gestational DM (ICD-9-CM 648.80–648.84).^[26]^ Patients who withdrew from the insurance program before the index date were also excluded. The control group consisted of randomly selected patients who never been diagnosed with thyroid disease and 4-fold size frequency matched with thyroid disease group by index year, age (every 5 years), and gender. The index date for the control group was randomly assigned a date between 2000 and 2012, and the exclusion criteria were the same as thyroid disease group.

### 2.3 Event and comorbidities

The event we discussed in this study was T2D (ICD-9-CM 250.x1, 250.x2). The end of the study was defined by occurrence of event, withdrawal from the insurance program, or the end of 2013. Comorbidities considered were hypertension (ICD-9-CM 401–405), dyslipidemia (ICD-9-CM 272), and obesity (ICD-9-CM 278).

### 2.4 Statistical analysis

We described the continuous variable age by mean and standard deviation (SD) and tested the difference between the thyroid disease group and control group by using the *t* test. The categorical variables were presented as number and percentage and by testing the difference by using the Chi-squared test. The follow-up time was the period from the index date to the end date; person-years were the sum of the follow-up time for each individual. The incidence rate of event was the number of events divided by the person-years. The hazard ratio (HR) and 95% confidence interval (95% CI) were estimated by univariable and multivariable Cox proportional hazard regression models. Variables of the multivariable model were gender, age, and all comorbidities. In this study, the primary analysis was of the thyroid disease group compared with control group; the second analysis was a comparison of the hyperthyroidism subgroup, hypothyroidism subgroup, and control group. We described the cumulative incidence of T2D for groups by use of the Kaplan–Meier method and testing the difference of groups by using the log rank test. The data analysis for this study was performed using SAS statistical software (Version 9.4 for Windows; SAS Institute, Inc., Cary, NC). *P* values less than 0.05 were considered statistically significant.

## 3 Results

**Figure 1:**
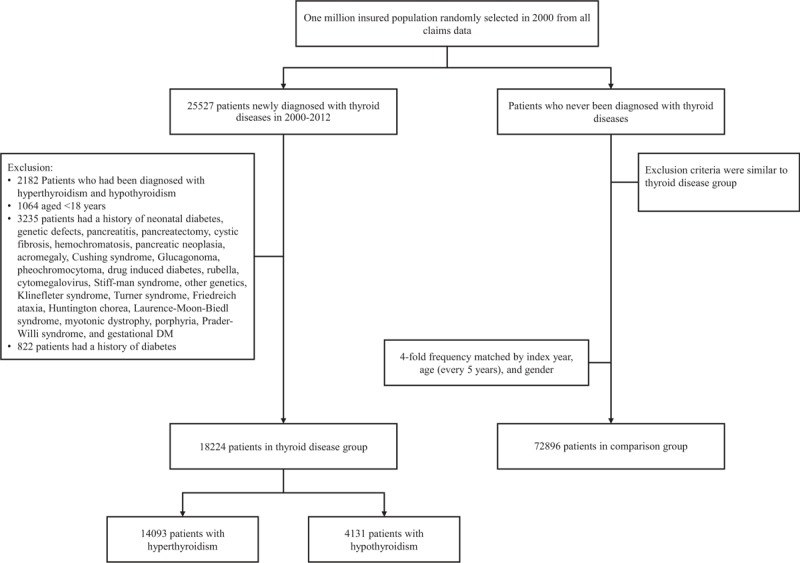
Flowchart shows the enrolment of the participants in the study cohort.

The study flowchart is shown in Fig. 1. In total, there were 18,224 patients with thyroid disease in this cohort. Of the total, 14,093 patients had hyperthyroidism and 4131 patients had hypothyroidism (Table 1). The control group with 4-fold frequency matching numbered 72,896 patients. Female patients numbered 56,456 in the control group and 14,114 in the thyroid disease group. There were 30,560 patients in the control group and 7640 patients in the thyroid disease group of an age between 40 and 64 years (y/o). There was no statistic difference between the 2 groups in demographic distribution.

**Table 1:**
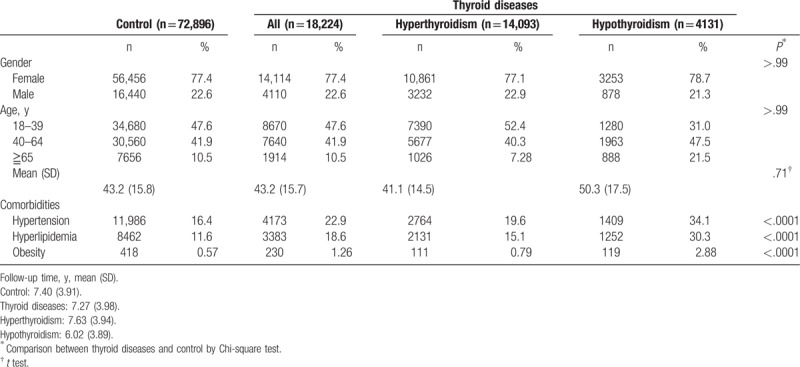
Baseline characteristics of patients.

There were 16.4% (11,986) in the control group and 22.9% (4173) in thyroid disease group with hypertension comorbidity. There were 11.6% (8462) with dyslipidemia in the control group, which was significantly lower than in the thyroid disease group, which represented 18.6% (3383). Comorbidity of obesity was also significantly lower in the control group (0.57%) as compared with the thyroid disease group (1.26%) (Table 1).

**Figure 2:**
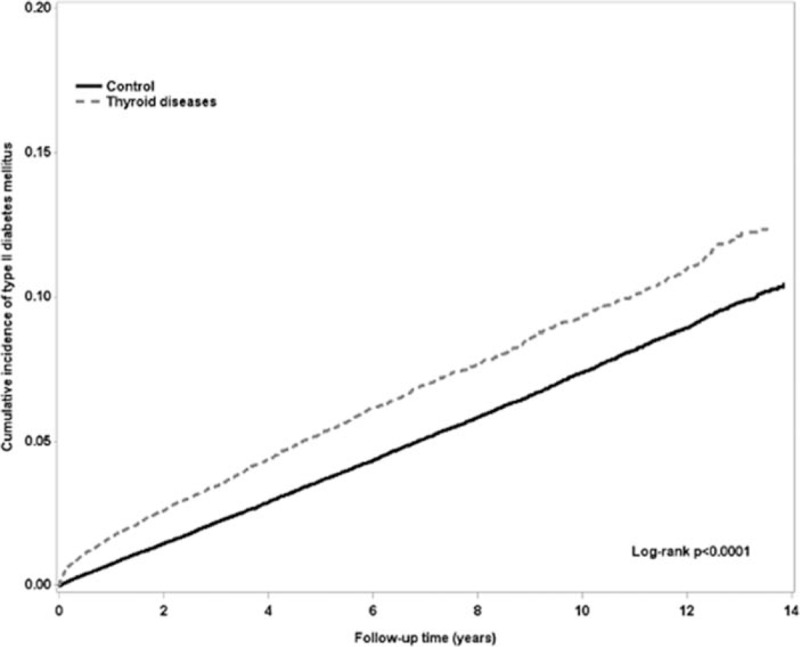
The cumulative incidence of type 2 diabetes mellitus between control and thyroid diseases patients.

Overall, the incidence rate and HR of T2D is summarized in Table 2. There was significantly higher occurrence of T2D in the thyroid disease group than in the control group, with HR of 1.23 (95% CI = 1.16–1.31) during the 10-year follow-up period. This was also seen in both the hyperthyroidism and hypothyroidism subgroups, with HR of 1.24 and 1.19, respectively. The cumulative incidence of T2D in patients with thyroid disease was higher than the control group (Fig. 2).

**Table 2:**
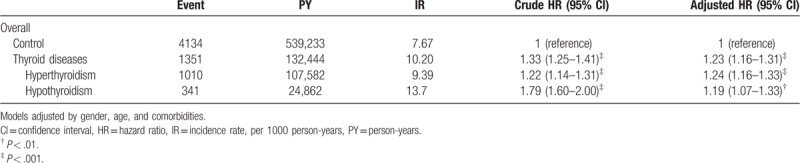
Incidence rate and hazard ratio of type 2 diabetes mellitus between control and thyroid diseases.

**Figure 3:**
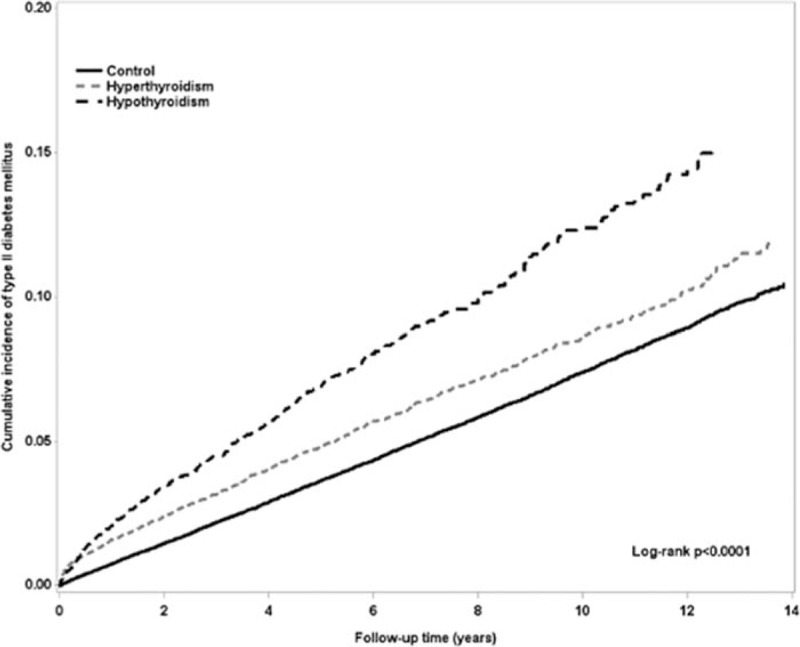
The cumulative incidence of type 2 diabetes mellitus between control, hyperthyroidism, and hypothyroidism patients.

After stratifying the study group by age and gender, female patients revealed a significantly higher occurrence of T2D in the thyroid disease group than the control group, with HR of 1.27 (95% CI = 1.18–1.37) (Table 3). There was significantly higher occurrence of T2D in the age category of 18 to 39 y/o and 40 to 64 y/o subgroups in the thyroid disease group than the control group. Higher occurrence of T2D was also seen in thyroid disease patients without comorbidity than in the control group, with HR of 1.47 (95% CI = 1.34–1.60). Cumulative incidence of T2D in both hyperthyroidism and hypothyroidism patients were higher than the control (Fig. 3, log rank *P* < .0001).

**Table 3:**
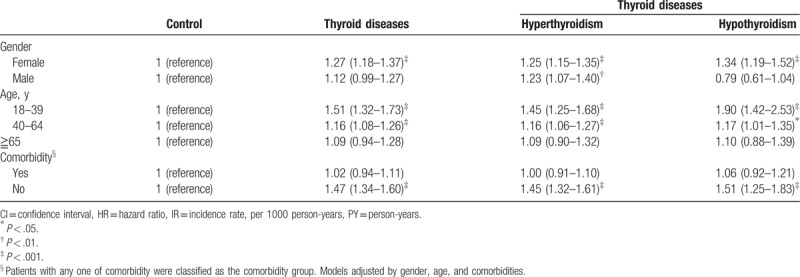
Adjusted hazard ratio of type 2 diabetes mellitus between control and thyroid diseases stratified by gender and age.

Table 4 depicts follow-up time of T2D between the thyroid disease and control groups. Patients with thyroid diseases had the most significantly higher chance of developing T2D than the control group within half a year follow-up period, with HR of 2.57 (95% CI = 2.15–3.08). Both hyperthyroidism and hypothyroidism revealed a higher chance, with HR of 2.78 and 2.12, respectively. Thyroid disease also revealed a higher chance of developing T2D within half to 1-year follow-up with HR of 1.39 (95% CI = 1.10–1.75). Hyperthyroidism revealed high HR (1.46) to develop T2D. Hypothyroidism, however, did not show statistical significance. Hyperthyroidism suggested an additional higher chance of developing T2D within 2 to 4-year follow-up, with HR of 1.21 (95% CI = 1.04–1.41). After further analyzing the long-term follow-up period of over 4 years, we found that the chance was equal to the control group.

**Table 4:**
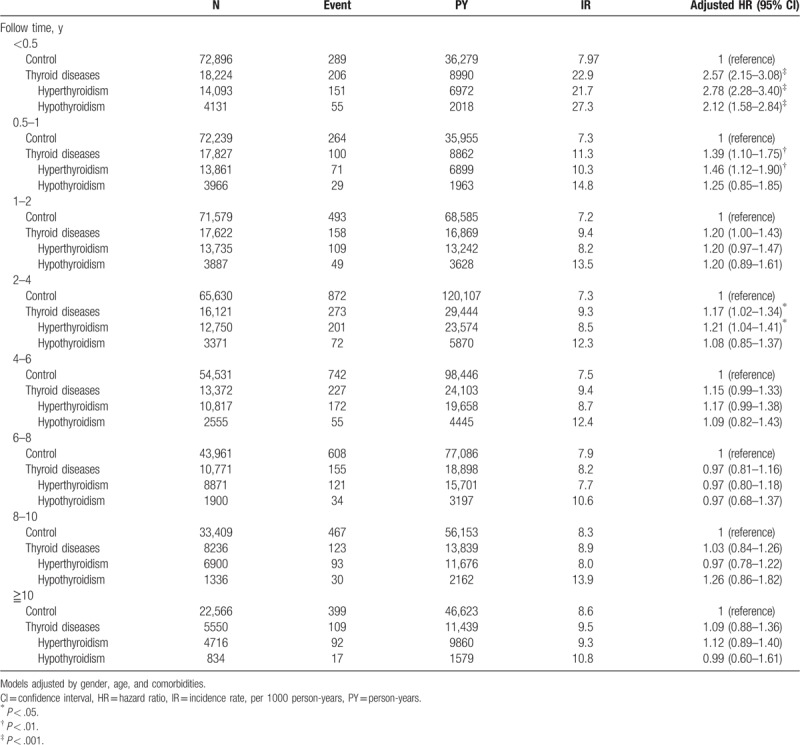
Incidence and hazard ratio of type 2 diabetes mellitus stratified by follow-up time.

## 4 Discussion

There was an association made between thyroid dysfunctions and DM. Patients with thyroid dysfunctions had significantly increased incidence of T2D in the 10-year follow-up period of this study. After considering gender and age, it was found that female patients and patients younger than 65 y/o had a higher risk of developing T2D. The results provide important clinical information that one should check blood sugar in patients who have been diagnosed with thyroid dysfunction, whether hyperthyroidism or hypothyroidism.

Thyroid hormones regulate the metabolism of carbohydrates. Therefore, thyroid dysfunctions may result in abnormality of carbohydrate metabolism and subsequent hyperglycemia. However, the incidence of DM in patients with hyperthyroidism was only 2% to 3.3% in an earlier report. There was also no association between hyperthyroidism and DM development reported in the contemporary literature.^[27]^ In a study that followed a total of 8452 pre-diabetic patients from the Netherlands for 7.9 years, the risk of DM occurrence was lower (0.96) in subjects with higher serum-free T4 level compared with the normal control.^[21]^ In addition, hypothyroidism patients possessed a higher risk (HR: 1.13–1.75) of developing DM than the controls from the above study and in an Israel study of around 60,000 patients.^[21,22]^ The inconsistent results have also been seen in other smaller serial studies.^[20,24,27]^ Nevertheless, our 10-year results suggest that both hyperthyroidism and hypothyroidism patients had higher cumulative rates of T2D occurrence than the control group.

After excluding the influence of sex, age, and comorbidities, risk ratios of T2D occurrence in both hyperthyroidism and hypothyroidism patients were still higher than the control group in our study. Thyroid dysfunctions suggested that interfering with the metabolism of carbohydrates promoted the development of DM. Possible mechanisms include insulin resistance and abnormal secretion and clearance of insulin.^[8,10,12–15,17]^

In subgroup analysis, we found that female patients with either hyperthyroidism or hypothyroidism were more prone to developing T2D than the control group. In male patients, the higher occurrence tendency of T2D was only seen in the hyperthyroidism patients. A possible explanation is the limited male patient number in our study or that male patients were less commonly seen with thyroid disease. With regard to age, the younger patients (<65 y/o) with thyroid dysfunctions were more prone to developing T2D than the control group. However, older patients (≧65 y/o) with thyroid dysfunctions had a likelihood of developing T2D similar to the control group. This may be due to insulin resistance and abnormities of insulin secretion, both generally caused by age. Therefore, the influence of thyroid dysfunctions on carbohydrate metabolism became relatively weak.^[28,29]^ This phenomenon was also seen in patients with comorbidities.

In our study, most patients with T2D were found to occur in the period of 6 months and 1 year after the presentation of hypothyroidism and hyperthyroidism, respectively. The occurrence rate of T2D in thyroid dysfunction patients decreased thereafter. This is due to the treatment of thyroid dysfunctions possibly improving thyroid function and reducing the occurrence of T2D. Interestingly, patients with hyperthyroidism also suggested higher occurrence rate of T2D 2 to 4 years after initial diagnosis. This may be due to recurrent hyperthyroidism, which is prone to occur within 2 years after stopping a course of antithyroid drug treatment (1 to 2 years), in some patients.^[30–32]^

Gronich et al reported that hypothyroidism was frequently seen and was a risk factor in new-onset T2D.^[22]^ They recommended that thyroid function tests should be performed in new-onset T2D. From our clinical observation, there seems to be a trend of previous thyroid disease increasing the incidence of T2D. We found that thyroid disease and T2D coexisted in some members among families. The age of onset of thyroid disease was younger than T2D, which were 44 and 47 y/o, respectively.^[33]^ Therefore, we believe that patients with previous thyroid disease indicate T2D in the future.

There were some limitations in this study. T2D is a commonly seen disease that may be related to many clinical factors, such as aging, family traits, and others. Those factors may contribute to the onset of T2D. We excluded other causes of DM in this cohort study to account for other possibilities that relate to T2D. However, there are some factors that could not be ruled out completely. Therefore, the factor of thyroid disease contributing to T2D may just be a small proportion of the patient population in which the results showed weak association. Although age of onset of T2D is 3 years older than thyroid disease, the 10-year study period shows a different tendency. However, a 10-year cohort study may not represent all disease onset related to thyroid disease. In addition, this cohort study was based on the database of the NHI, which did not include biochemical data of patients to evaluate the severity of disease. We try as far as possible in this study to recognize the development of true T2D. However, we could not completely exclude the possibility of type 1 DM (T1D) occurrence in both groups of subjects. T1D is an autoimmune-related disease and may coexist with Graves disease and chronic thyroiditis, the major causes of hyperthyroidism and hypothyroidism, respectively. In addition, in subjects with hypothyroidism, unlike patients with hyperthyroidism who always present prominent and impressive symptoms/signs, the onset of thyroid dysfunction is usually insidious and the clinical manifestations may go unnoticed by patients themselves, family, friends, and clinicians, even endocrine specialists.

## 5 Conclusion

There was a relatively high risk of T2D development in patients with thyroid dysfunctions, especially in the period of 0.5 to 1 year after presentation of thyroid dysfunctions. For early diagnosis and appropriate treatment of DM, blood sugar tests should be performed in such patients after the initial diagnosis of thyroid dysfunctions. On the contrary, it is also necessary to re-evaluate the status of blood sugar and to consider long-term antidiabetic treatment or not once thyroid function restore to normal.

## Author contributions

**Conceptualization:** Huey-Yi Chen, Wen-Chi Chen.

**Data curation:** Weishan Chen, Yung-Hsiang Chen.

**Funding acquisition:** Wen-Chi Chen, Yung-Hsiang Chen.

**Investigation:** Rong-Hsing Chen, Huey-Yi Chen, Kee-Ming Man, Szu-Ju Chen, Weishan Chen, Po-Len Liu, Wen-Chi Chen, Yung-Hsiang Chen.

**Methodology:** Rong-Hsing Chen, Huey-Yi Chen, Kee-Ming Man, Szu-Ju Chen, Weishan Chen, Po-Len Liu, Wen-Chi Chen, Yung-Hsiang Chen.

**Project administration:** Rong-Hsing Chen, Wen-Chi Chen, Yung-Hsiang Chen.

**Resources:** Kee-Ming Man, Wen-Chi Chen.

**Supervision:** Wen-Chi Chen, Yung-Hsiang Chen.

**Validation:** Rong-Hsing Chen, Huey-Yi Chen, Kee-Ming Man, Wen-Chi Chen.

**Writing – original draft:** Rong-Hsing Chen, Wen-Chi Chen.

**Writing – review & editing:** Wen-Chi Chen, Yung-Hsiang Chen.

Yung-Hsiang Chen orcid: 0000-0002-8756-5113.
